# Impact of Biotic/Abiotic Stress Factors on Plant Specialized Metabolites

**DOI:** 10.3390/ijms25115742

**Published:** 2024-05-25

**Authors:** Maciej Strzemski, Sławomir Dresler

**Affiliations:** 1 Department of Analytical Chemistry, Medical University of Lublin, Chodźki 4a Street, 20-093 Lublin, Poland; 2 Department of Plant Physiology and Biophysics, Institute of Biological Sciences, Faculty of Biology and Biotechnology, Maria Curie-Sklodowska University, Akademicka 19 Street, 20-033 Lublin, Poland

Plants are a group of organisms that have developed remarkable adaptations to merely exist in the environment. Lacking the ability to move, escape, or actively fight predators, these organisms have had to develop mechanisms for chemical adaptation and defense against harmful environmental conditions and the effects of phytophages. The mutualistic bio-chemical interactions of plants with plants, insects, bacteria, viruses, and fungi, as well as chemical agents (including heavy metal ions, toxic gases, xenobiotics, and microplastics), physical agents (including UV and ionizing radiation, low and high temperatures, and mechanical damage), and mixed agents (radioisotope ions, high salinity, excess or deficiency of water, osmotic stress) occurring in the environment, have long been the subject of countless research (Figure 1). Despite the ever-growing body of knowledge, these issues will continue to remain relevant. Taking into account the abundance of species in the plant kingdom (totaling about 300,000) and organisms that compete with them, as well as the diversity of plant habitats, climate change, and anthropopressure, it can be concluded that this Special Issue is an inexhaustible resource that will provide researchers with enormous cognitive opportunities. The results of investigations into the effects of various stress factors on plant metabolism provide a better understanding of plant physiology—the functioning of secondary metabolite biosynthesis pathways, detoxification of xenobiotics, and mechanisms of adaptation to adverse conditions that naturally occur in the environment. The knowledge thus gained creates the possibility to uncover more complex relationships at the level of ecosystem functioning in ecological and evolutionary terms. This research also has tremendous application value, creating an opportunity for the following, among other possibilities: the control of phytochemical biosynthesis [1], plant habitats [2,3], and pest populations [4,5]; the reclamation of anthropogenically altered areas [6]; phytomining [7], and the extraction of significant amounts of metabolites from plants growing in these areas [8]. Thus, studying plant interactions with biotic and abiotic stress factors is fundamental to many fields of science, both basic and applied.

Human activities and the enormous pressures on the environment that are associated with them are leading to multifactorial adverse effects that disrupt normal plant growth and development. Given the combined effect of multiple stresses on plant physiology, it is important to adopt a comprehensive approach that recognizes the complexity of the environment [9,10]. In this context, questions arise as to the possible interactions between stress factors and the synergistic or antagonistic effects of stress factors on plants, the acclimatization and adaptation of plants to multifactorial stress, and the mechanisms of plant responses to multifactorial stress [9,10]. These issues represent extremely important scientific challenges that, in times of global environmental change, including climate change, global warming, and increasing environmental pollution, introduce obstacles in mitigating problems such as crop production or the decreasing biodiversity of natural ecosystems [11,12].

Six original articles are published in this Special Issue, the topics of which reflect the extremely broad-spanning issue of the effect of stress on the biosynthesis and accumulation of metabolites of specialized plant metabolism. We are confident that these papers are excellent contributions to the development of knowledge on the effects of biotic and abiotic stresses on specialized plant metabolism and will pave the way for further research in this area.

## Figures and Tables

**Figure 1:**
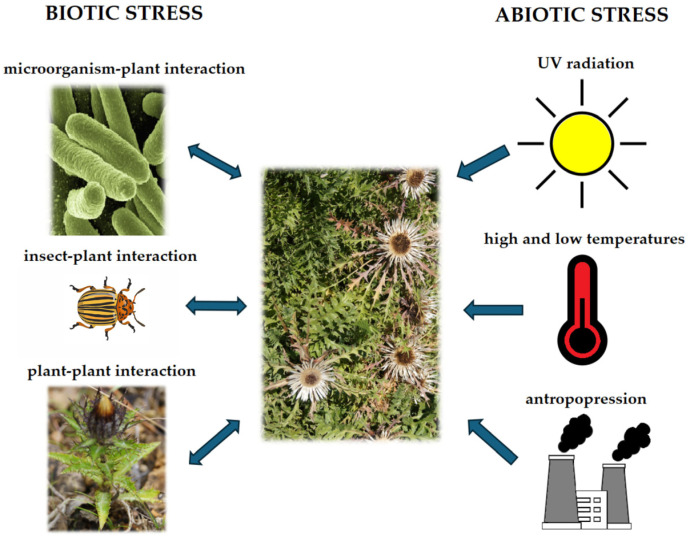
The main biotic and abiotic stresses affecting plants.

## References

[1] Hanaka A., Majewska M., Jaroszuk-Ściseł J. (2022). Study of the influence of abiotic and biotic stress factors on horticultural plants. Horticulturae.

[2] Khamare Y., Chen J., Marble S.C. (2022). Allelopathy and its application as a weed management tool: A review. Front. Plant Sci..

[3] Trezzi M.M., Vidal R.A., Junior A.A.B., von Hertwig Bittencourt H., da Silva Souza Filho A.P. (2016). Allelopathy: Driving mechanisms governing its activity in agriculture. J. Plant Interact..

[4] Spinozzi E., Ferrati M., Cappellacci L., Caselli A., Perinelli D.R., Bonacucina G., Maggi F., Strzemski M., Petrelli R., Pavela R. (2023). *Carlina acaulis* L. (Asteraceae): Biology, phytochemistry, and application as a promising source of effective green insecticides and acaricides. Ind. Crops Prod..

[5] Farooq M., Jabran K., Cheema Z.A., Wahid A., Siddique K.H. (2011). The role of allelopathy in agricultural pest management. Pest Manag. Sci..

[6] Peco J.D., Higueras P., Campos J.A., Esbrí J.M., Moreno M.M., Battaglia-brunet F., Sandalio L.M. (2021). Abandoned mine lands reclamation by plant remediation technologies. Sustainability.

[7] Kikis C., Thalassinos G., Antoniadis V. (2024). Soil Phytomining: Recent Developments—A Review. Soil Syst..

[8] Dresler S., Rutkowska E., Bednarek W., Stanisławski G., Kubrak T., Bogucka-Kocka A., Wójcik M. (2017). Selected secondary metabolites in *Echium vulgare* L. populations from nonmetalliferous and metalliferous areas. Phytochemistry.

[9] Zandalinas S.I., Peláez-Vico M.Á., Sinha R., Pascual L.S., Mittler R. (2024). The impact of multifactorial stress combination on plants, crops, and ecosystems: How should we prepare for what comes next?. Plant J..

[10] Zandalinas S.I., Mittler R. (2022). Plant responses to multifactorial stress combination. New Phytol..

[11] Majumder D., Saha S., Chandran M.A.S., Bal S.K., Pathak H., Chatterjee D., Saha S., Das B. (2024). Prospects of Modified Plant Micro-Climate in Global Climate Change Research. Climate Change Impacts on Soil-Plant-Atmosphere Continuum.

[12] Hasanuzzaman M. (2020). Plant Ecophysiology and Adaptation under Climate Change: Mechanisms and Perspectives II: Mechanisms of Adaptation and Stress Amelioration.

